# Hematite Nanoparticles from Unexpected Reaction of Ferrihydrite with Concentrated Acids for Biomedical Applications

**DOI:** 10.3390/molecules25081984

**Published:** 2020-04-23

**Authors:** Afanasy V. Lunin, Anna A. Lizunova, Elizaveta N. Mochalova, Maria N. Yakovtseva, Vladimir R. Cherkasov, Maxim P. Nikitin, Eugene L. Kolychev

**Affiliations:** 1 Moscow Institute of Physics and Technology, 9 Institutskiy per., Dolgoprudny, 141700 Moscow Region, Russia; afanasy.lunin@phystech.edu (A.V.L.); anna.lizunova@gmail.com (A.A.L.); mochalova@phystech.edu (E.N.M.); mariantes@yandex.ru (M.N.Y.); v_r_cherkasov@list.ru (V.R.C.); max.nikitin@phystech.edu (M.P.N.); 2 Prokhorov General Physics Institute of the Russian Academy of Sciences, 38 Vavilova St., 119991 Moscow, Russia; 3 Shemyakin–Ovchinnikov Institute of Bioorganic Chemistry, Russian Academy of Sciences, Miklukho-Maklaya St., 16/10, 117997 Moscow, Russia

**Keywords:** hematite nanoparticles, nanoparticle synthesis, ferrihydrite, polymer coating, bioconjugation, antibodies, red blood cells, HER2/neu, bioimaging, cancer cell targeting

## Abstract

The development of synthetic ways to fabricate nanosized materials with a well-defined shape, narrow-sized distribution, and high stability is of great importance to a rapidly developing area of nanotechnology. Here, we report an unusual reaction between amorphous two-line ferrihydrite and concentrated sulfuric or other mineral and organic acids. Instead of the expected dissolution, we observed the formation of new narrow-distributed brick-red nanoparticles (NPs) of hematite. Different acids produce similar nanoparticles according to scanning (SEM) and transmission electron microscopy (TEM), selected area electron diffraction (SAED), X-ray diffraction (XRD), infrared spectroscopy (FTIR), and energy-dispersive X-ray spectroscopy (EDX). The reaction demonstrates new possibilities for the synthesis of acid-resistant iron oxide nanoparticles and shows a novel pathway for the reaction of iron hydroxide with concentrated acids. The biomedical potential of the fabricated nanoparticles is demonstrated by the functionalization of the particles with polymers, fluorescent labels, and antibodies. Three different applications are demonstrated: i) specific targeting of the red blood cells, e.g., for red blood cell (RBC)-hitchhiking; ii) cancer cell targeting in vitro; iii) infrared ex vivo bioimaging. This novel synthesis route may be useful for the development of iron oxide materials for such specificity-demanding applications such as nanosensors, imaging, and therapy.

## 1. Introduction

The reaction of freshly precipitated ferrihydrite with dilute sulfuric acid is an important textbook example of the basicity of iron (III) [1]. Moreover, this reaction is of interest to scientists working in the area of planetary chemistry as it is proposed to be occurring on Mars during asteroid impacts [2] and could take place on Venus [3]. This reaction is thought to result in a quick dissolution of the precipitate with a formation of nonturbid transparent solution of iron (III) sulfate. It was reported that the reaction of ferrihydrite with diluted acids lasts for months or even years, leading to the formation of hematite and goethite micro- and nanoparticles [1,4,5,6,7,8,9,10,11]. Additionally, the preparation of hematite nanoparticles can be achieved by forced hydrolysis of iron (III) salts [12], hydrothermal processes [13,14], and by green chemistry methods [15,16]. Recently, the authors reported on the rapid and facile synthesis of hydrous ferric oxide nanoparticles by the nitric acid-induced transformation of ferrihydrite [17]. Due to the generally low toxicity of hematite nanoparticles [18,19], such materials are promising for different areas of biomedicine. Moreover, the nanoparticles with controllable shape and narrow size distribution could be used as building blocks in most demanding and advanced applications to date, such as the development of “smart” agents for in vitro diagnostics [20,21] and drug delivery [22,23,24]. Therefore, we attempted to investigate this synthesis more carefully and observed an unusual reaction between concentrated acids and ferrihydrite. The reaction led to the formation of near-monodisperse hematite nanoparticles instead of their full dissolution.

## 2. Results and Discussion

We became intrigued by the idea that the formation of hematite and other iron oxides instead of full mineral dissolution is possible in strongly acidic environments. Therefore, an investigation into the fate of ferrihydrite on Venus, Early Earth, or Mars during intense asteroid impacts was conducted. Lower temperatures and the sulfuric acid, abundant under the conditions, were used as critical factors in our preliminary model system. The interaction between ferrihydrite and 35% *w/w* sulfuric acid solution at the typical temperature on Mars (−60 °C) was studied to model the natural conditions on that planet. The ferrihydrite was synthesized via the reaction between aqueous solutions of ferric chloride and aqueous ammonia; ferrihydrite water-based paste was prepared by the centrifugation of its suspension.

**Figure 1:**
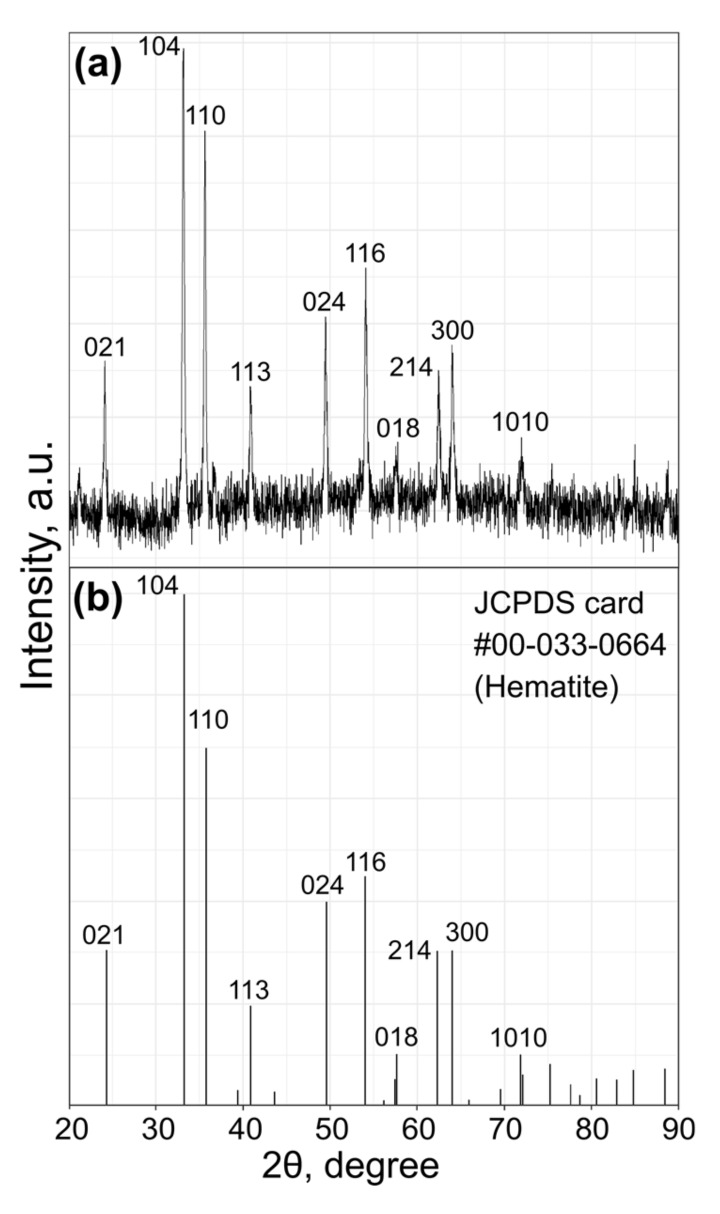
(**a**) The X-ray diffraction patterns of the synthesized nanoparticles; (**b**) The reference patterns.

After the addition of the paste to the sulfuric acid solution, the color of the mixture immediately turned brick-red. Samples were collected during the reaction for analysis and, surprisingly, a few drops of the solution left on the pipette remained turbid and brick-red for at least several hours at room temperature. Only after two days, the mixture turned into a non-turbid and transparent solution, which indicated the formation of ferric sulfate. Therefore, we set a similar reaction at a higher temperature (0–4 °C) with the concentrated sulfuric acid (98% *w/w*). The reaction was carried out as follows: the sulfuric acid was cooled with an ice bath, and upon vigorous stirring, ferrihydrite paste was added to it, stirred for 5 min and quenched by pouring into an excess of ice (for the experimental setup, see Figure S1). The mixture was centrifuged twice and washed with water. X-ray diffraction (XRD) was used to analyze the sample. The XRD pattern shown in Figure 1 can be assigned to the phase of pure hematite α-Fe_2_O_3_ (JCPDS card: #00-033-0664) with no significant phase impurities. The paramagnetic nature of the obtained particles was verified by the magnetic particle quantification (MPQ) technique [25], which revealed no presence of ferro- and superparamagnetic fractions at the level of 0.1 ng/mL.

**Figure 2:**
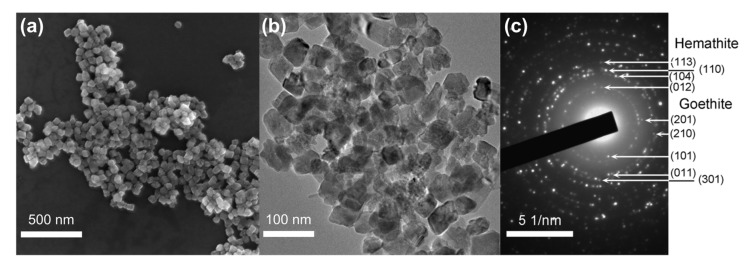
(**a**) Scanning electron microscopy; (**b**) Transmission electron microscopy images of the nanoparticles (NPs); (**c**) Selected area electron diffraction pattern of the NPs.

Scanning electron microscopy revealed that the precipitate consists of near cubic particles with ~50 nm size, as shown in Figure 2a. Further analysis by transmission electron microscopy (TEM) revealed more complex shapes than the cubic shape of the particles, similar to previously reported hematite nanocrystals, as shown in Figure 2b, and Figure S2 [26,27]. The energy-dispersive X-ray spectroscopy (EDX) analysis showed that the nanoparticles (NPs) contain no detectable traces of other elements, as shown in Figure S3. The atomic ratio of Fe:O in the sample is about 1:2.8, which is lower than it is expected for hematite due to low accuracy of the method in the routine analysis of light elements (z < 11). Therefore, a standard sample of hematite prepared by a well-established textbook procedure [1] and the EDX analysis showed the same ratio of Fe:O, as presented in Figure S4.

**Figure 3:**
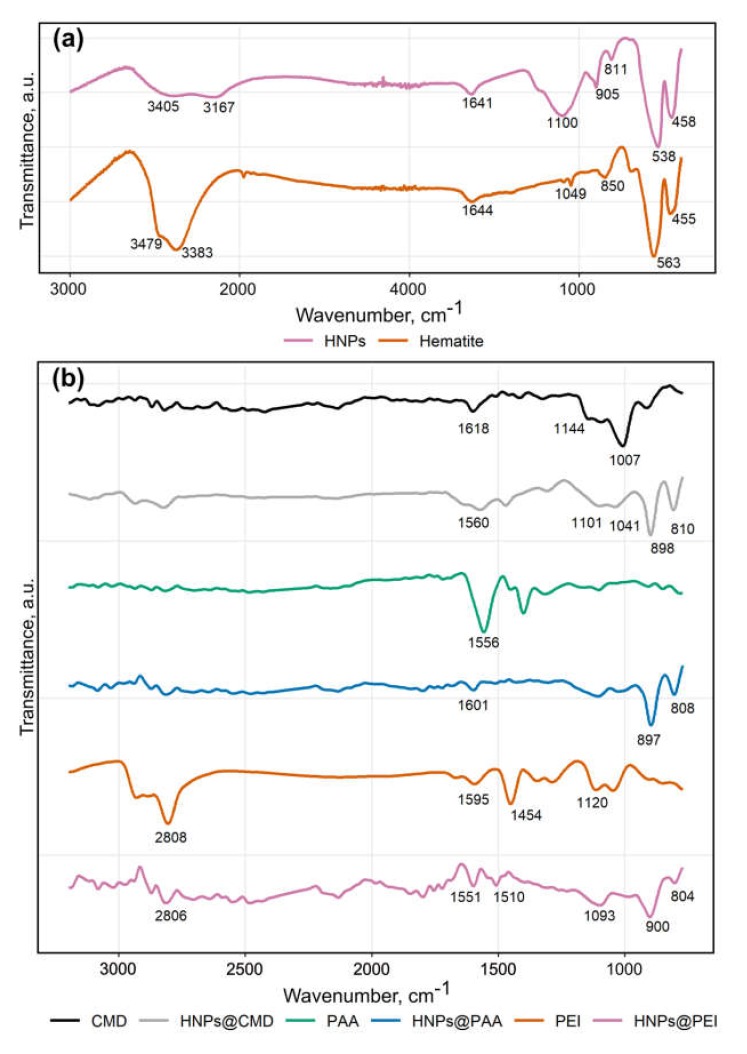
Attenuated total reflectance (ATR) infrared spectroscopy (FTIR) spectra. (**a**) Comparison of the hematite nanoparticles (HNPs) spectrum with the standard hematite sample; (**b**) Spectra of polymer-coated HNPs in 770–3200 cm^−1^ range in comparison with the spectra of pure polymers.

The crystalline structure of the particles was determined using the selected area electron diffraction analysis (SAED), as shown in Figure 2c. The SAED pattern exhibits bright rings corresponding to a large amount of hematite nanocrystals and spot reflexes that refer to single particles of goethite. Some of the main lattice spacings are similar to hematite or goethite ones. The only way to distinguish these phases in the electron diffraction patterns is the presence of a ring with a lattice spacing of 0.369 nm corresponding to the (012) plane of hematite. This ring is located near to the central beam spot and several single spots with weak intensity obtained from goethite plane (101) and plane (201) (with interplanar spacing of 0.416 and 0.336 nm, respectively). Therefore, the NPs consist of a mixture of hematite (α-Fe_2_O_3_) and traces of goethite (α-FeO(OH)). The attenuated total reflectance infrared spectroscopy (FTIR) spectrum of the prepared NPs contains peaks identical to those in the spectrum of the standard hematite sample as shown in Figure 3a, and also contains peaks at 905 cm^−1^ and 811 cm^−1^ characteristic of hydrated iron oxide species such as goethite [RRUFF ID: R050142.1]. Since the FTIR analysis in the reflectance mode shows mostly the nature of the surface of the NPs and does not depend on the crystallinity of the analyzed compound and, taking into account the XRD results, we could assume that the nanoparticles consist of a hematite core with a thin layer of mostly amorphous hydrated iron oxide on the surface.

The reaction yield was about 5%; therefore, a possibility to increase the yield was studied. A variety of concentrated acids was examined to compare their ability to form hematite nanoparticles (HNPs), as shown in Figure S5 and Figure S6. In the case of the concentrated nitric acid, the similar HNPs were formed with a much lower yield; similarly, the precipitate was stable in the acid for at least a couple of days.

The hydrochloric acid produced the same hematite precipitate, which dissolved completely in less than 30 min. Organic acids, such as pure formic and glacial acetic acids, again formed small amounts of hematite NPs (~1%). However, 24-h stirring at room temperature was required to complete the reaction, which was monitored by a color change from dark brown to brick-red. On the other hand, concentrated phosphoric acid provided the result similar to sulfuric acid with the same yield of 5%.

It was assumed that the formation of the HNPs depends not only on the strength of acid but also on its dehydrating ability. Therefore “100% phosphoric acid” (known to be an equilibrium mixture of phosphoric acid, pyrophosphoric acid, and water) was used, and the yield of NPs obtained was almost twice high (9%). The same result (10%) was obtained using a mixture of sulfuric acid and phosphorous pentoxide as well. Therefore, strong acidic dehydrating agents are capable of transforming ferrihydrite to less hydrated ferric oxides instead of dissolving them. Since ferrihydrite paste used in the experiments had water content that could lead to a dilution of the acid and a decrease in its dehydrating ability in one experiment, water was replaced with ethanol, which, however, did not lead to an increased yield of the NPs. Finally, a scale-up experiment was carried out using c.a. 10-fold quantities of the starting reagents and the mixture of the sulfuric acid and phosphorous pentoxide. The same NPs were obtained in 9.7% yield (0.51 g).

To evaluate the potential of the HNPs for biomedical applications, a few polymers were used to create a surface functional layer for further facile bioconjugations. Polymers containing carboxylic groups, such as carboxymethyldextran sodium salt (CMD, Mn ~ 15,000), polyacrylic acid sodium salt (PAA, Mn ~ 5100), as well as polyethyleneimine (PEI, Mn ~ 25,000) containing amino groups, were immobilized onto the surface of the NPs using a straightforward procedure of incubation with the excess of polymers in hot aqueous solutions. The hydrodynamic radii of the NPs determined by dynamic light scattering (DLS) analysis were 65 ± 9 nm, 62 ± 8 nm, and 76 ± 8 nm for HNPs@CMD, HNPs@PAA, and HNPs@PEI, respectively (the DLS data are presented in Figure S7). The radii were slightly decreased from 90 ± 8 nm for the HNPs, demonstrating higher stabilization of the NPs by polymers against aggregation. The presence of the organic coating was also confirmed by FTIR spectroscopy, as shown in Figure 3b. In the case of CMD and PAA coating, the characteristic peaks of CMD (1618 cm^−1^ and 1560 cm^−1^) and PAA (1556 cm^−1^ and 1601 cm^−1^) carboxylic groups broadened and slightly shifted due to presence of nonequivalent groups non-coordinated and coordinated to the iron oxide surface. Similarly, a more complex pattern was observed for PEI after coordination to the NPs surface.

**Figure 4:**
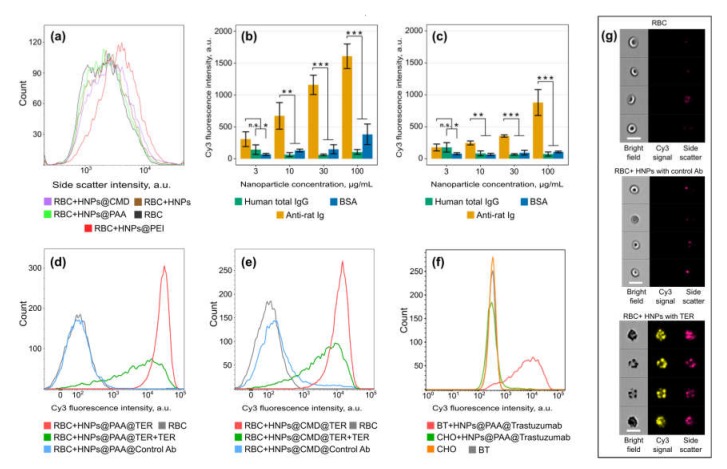
Flow cytometry and fluorescent immunoassay results. (**a**) Interaction between erythrocytes (RBCs) and coated HNPs, higher side scattering indicates stronger interactions; (**b**), (**c**) Binding of HNPs@PAA@TER-Cy3 (**b**) and HNPs@CMD@TER-Cy3 (**c**) with human total immunoglobulin (IgG), bovine serum albumin (BSA), and anti-rat Ig, adsorbed on polystyrene, the data are plotted as mean  ± standard deviation (*n* = 3); (**d**), (**e**) Imaging flow cytometry analysis of the interaction between HNPs@PAA@TER-Cy3 and HNPs@CMD@TER-Cy3 with red blood cells (RBCs), green lines show the distribution obtained in the TER-119 monoclonal antibody (TER)-containing incubation mixture; (**f**) Flow cytometry analysis of the interaction of BT-474 (HER2/neu-positive) and CHO (HER2/neu-negative) cells with HNPs@PAA@Trastuzumab-Cy3; (**g**) Images of an interaction between RBCs and two types of polyacrylic acid sodium salt (PAA)-coated nanoparticles, conjugated with the RBC-binding and RBC-nonbinding antibodies (in bright field, Cy3-channel and in side scatter channel). The scale bar is 10 μm. Significance levels were calculated using unpaired one-tailed t-test (* *p* < 0.05; ** *p* < 0.01; *** *p* < 0.001; n.s. *p* > 0.05).

Firstly, the feasibility of nanoparticle functionalization with antibodies was investigated by flow cytometry and fluorescent immunoassay as displayed in Figure 4. The RBCs are a convenient model for the development of drug delivery agents because these cells lack endocytic machinery; thus, membrane–HNPs interaction analysis was not influenced by uptake [28]. The results showed that the nanoparticles modified with a positively charged PEI tend to nonspecifically bind to cells, while CMD and PAA did not, as shown in Figure 4a. Therefore, CMD- and PAA-coated HNPs were conjugated, using a carbodiimide-based conjugation procedure [29] with the TER-119 monoclonal antibody (TER) that recognizes erythroid-specific TER-119 antigens. The resulting HNPs@CMD@TER and HNPs@PAA@TER were labeled with fluorescent Cy3 fluorescent dye by treating the NPs with N-hydroxysulfosuccinimide ester of Cy3 (Cy3-sulfo-NHS). The efficiency of antibody coating and fluorescent labeling was evaluated with direct fluorescent immunoassay (FIA) [30]. In this experiment, goat anti-rat immunoglobulin (Ig), human total Ig and bovine serum albumin (BSA) were adsorbed on the polystyrene surface of 96-well microtiter plate followed by an addition of HNPs@CMD@TER-Cy3 and HNPs@PAA@TER-Cy3. After thorough washing, the efficiency of the fluorescent labeling was assessed by a fluorescent signal as shown in Figure 4b,c, that confirmed successful modification of both types of the HNPs which were bound specifically only to goat anti-rat Ig. HNPs@CMD@TER-Cy3 and HNPs@PAA@TER-Cy3 together with HNPs conjugated with the RBC nonbinding antibodies as a negative control were used in imaging flow cytometry studies as shown in Figure 4d,e to estimate nonspecific interaction of the HNPs. The results demonstrated higher specificity of PAA-modified HNPs to RBCs than CMD-modified HNPs. Additionally, the specificity of TER-conjugated HNPs was confirmed in competitive assay, using a free TER antibody together with the HNPs in solution during the incubation with cells. High binding efficiency of the TER-conjugated NPs with the RBCs was also confirmed by images obtained from imaging flow cytometry, as shown in Figure 4g. Thus, the TER-modified HNPs are promising candidates for successful drug delivery via RBC-hitchhiking in cancer therapy [22].

Next, high specificity of the agents for targeting cancer cells was demonstrated, as shown in Figure 4f. As the model for the targeting study, we chose BT-474 cells overexpressing HER2/neu epidermal growth factor receptor, a highly significant clinical cancer marker that is overexpressed in many types of human malignancies [31]. Chinese hamster ovary (CHO) cells were used as the HER2/neu-negative control. We used the anti-HER2/neu antibody trastuzumab, which was conjugated with the HNPs@PAA and labeled with Cy3. As can be seen from the figure, the trastuzumab-conjugated nanoparticles specifically targeted BT-474 cells, thus the NPs showed high specificity to HER2/neu receptor and can be used to target cancer cells. Accordingly, the nanoagents could be engineered for various applications (e.g., biosensorics or toxic agent removal) via functionalization with appropriate antibodies.

**Figure 5:**
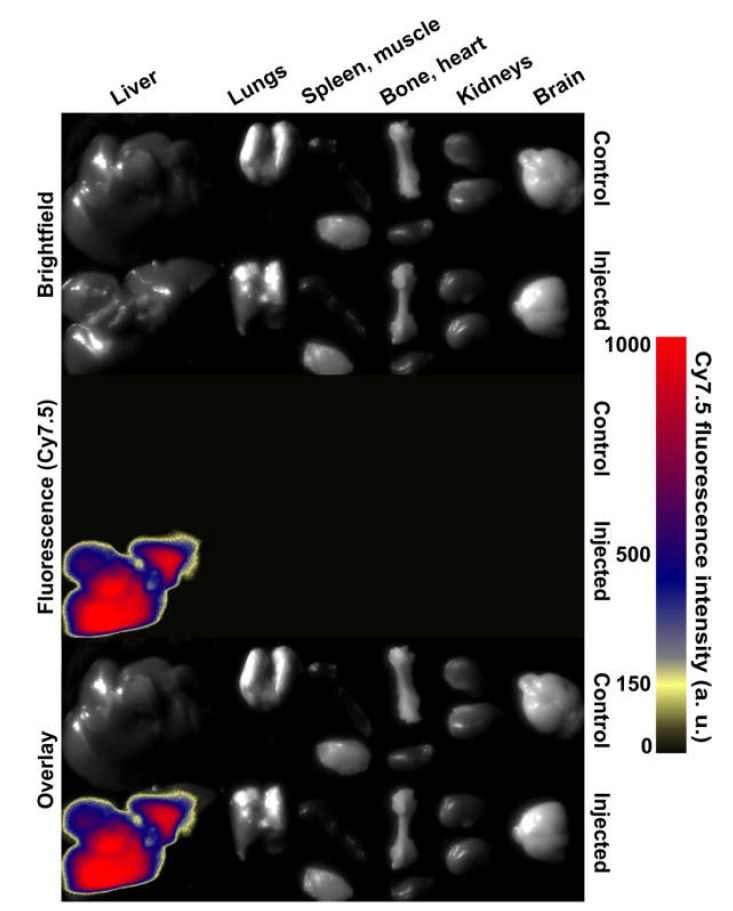
Fluorescent ex vivo imaging of the excised mouse organs.

Finally, we conjugated HNPs@PAA with total human IgG and labeled the conjugate with Cy7.5-sulfo-NHS (HNPs@PAA@IgG-Cy7.5) and studied its biodistribution. The NPs were injected into mice, and after 40-min incubation, the excised mouse organs were studied using infrared optical imaging, as shown in Figure 5. The fluorescent analysis showed that almost all the NPs were accumulated in the liver. This result may be explained by the existence of the liver-associated macrophage subpopulation that uptakes the NPs. This fact assumes that the NPs are proper agents for targeted drug delivery due to negligible nonspecific accumulation of the NPs in almost all organs (except the liver). Further studies will be focused on the receptor-specific delivery of the NPs into tumors and on adsorbing an agent for chemotherapy or photodynamic therapy on the surface of the NPs.

## 3. Materials and Methods

FeCl_3_·6H_2_O, Fe_2_(SO_4_)_3_·xH_2_O, P_4_O_10_, glacial acetic, pure formic acids, carboxymethyl-dextran sodium salt (CMD, >90%, cat. 86524-100G-F), 1-ethyl-3-(3-dimethylaminopropyl)carbodiimide hydrochloride (EDC, 98-100%), N-Hydroxysulfosuccinimide sodium salt (>99%), poly(acrylic acid sodium salt) average Mn ~ 5100 (cat. #447013-100G), polyethyleneimine 25,000 (>99%), and PBS were purchased from Sigma-Aldrich (St. Louis, MO, USA). MES buffer (>99%) was purchased from AppliChem,( Darmstadt, Germany). Nitric acid (70%, *w/w*), sulfuric acid (98% *w/w*), phosphoric acid (85%), aqueous ammonia (25% *w/w*), and bovine serum albumin (BSA, >99%) were purchased from Dia-M (Moscow, Russia). Cy3-sulfo-NHS and Cy7.5-sulfo-NHS esters were purchased from Lumiprobe, (Moscow, Russia). TER-119 (TER) antibodies were purchased from BioXCell, (Lebanon, NH, USA). Human total Ig (medicine grade) was obtained from Microngen (Moscow, Russia). Trastuzumab (Herticad) was purchased from Biocad (Moscow, Russia). Goat anti-rat antibodies were purchased from Jackson ImmunoResearch, (West Grove, PA, USA). All commercially available reagents were used as received. A standard hematite sample was prepared by forced hydrolysis of acidic aqueous FeCl_3_ solution [1]. Milli-Q water (Merck Millipore, Billerica, MA, USA) was used in the preparation of aqueous solutions. All the experimental animal procedures were performed in conformity with the Guide for the Care and Use of Laboratory Animals and approved by the Animal Care and Use Committee of the Institute of Bioorganic Chemistry, RAS, Moscow, Russia (protocol #240 of 01.01.2018).

Ferrihydrite was prepared by the reported procedure [32]. 100 mL of 0.33 M aqueous solution of ferric chloride hexahydrate (17.8 g of solid FeCl_3_·6H_2_O) was mixed with 25 mL of aqueous ammonia (25% *w/w*). The reaction mixture was kept stirring at 90 °C for 2 h, centrifuged at 1000× *g* for 1 min, washed 3 times with Milli-Q water followed by centrifugation at 1000× *g*.

The reaction of ferrihydrite and acids was carried out as follows. 10 mL of concentrated acid was cooled with an ice bath in 50 mL beaker with an X-shaped stirring bar as shown in Figure S1, then upon vigorous stirring, 3 mL of ferrihydrite paste (the concentration of dry content was 20 mg/mL) were added and after 5 min quenched by pouring into a 100 mL of ice. The volume of the ferrihydrite paste was measured with an open mouth syringe (1 cm in diameter). The centrifugation at 2300× *g* followed by repeated washing with ice-cold Milli-Q water resulted in the brick-red precipitate, which was washed with ethanol, diethyl ether, and dried overnight at ambient temperature. 100% phosphoric acid for the experiment was prepared by careful dissolution of 14 g P_4_O_10_ in 10 mL 85% phosphoric acid. The mixture of the sulfuric acid and P_4_O_10_ was prepared by dissolution of 5 g P_4_O_10_ in 10 mL of 98% sulfuric acid. For the scale-up experiment, 100 mL of the sulfuric acid and 50 g P_4_O_10_ were used. In this case, the reaction was carried out in a 250 mL round-bottom flask equipped with a 2 × 4 cm olive-shaped magnetic stirring bar.

Chinese hamster ovary (CHO) and BT-474 cell lines were cultured in DMEM/Ham’s F12 (1:1) essential media (PanEco, Moscow, Russia), containing 100 units/mL penicillin-streptomycin (PanEco) and supplemented with 10% fetal bovine serum (Hyclone, Logan, UT, USA ) and 300 mg/L L-glutamine (PanEco). An incubator with 5% CO_2_ was used to culture the cells at 37 °C. The harvested cells were kept on ice in PBS prior to the experiment.

To isolate the red blood cells (RBCs), heparinized mouse (BALB/c) whole blood was centrifuged for 5 min at 500× *g* and washed with PBS 3 times. Then, the RBCs were kept on ice in PBS prior to the experiment.

Coating of the nanoparticles with different polymers by combination of donor–acceptor and electrostatic interactions between functional groups of polymers and iron oxide surface of NPs was performed using the identical procedure. 10 mg of dry HNPs were sonicated with 1 mL of 0.1 M HCl, washed with Milli-Q water with subsequent centrifugation three times and mixed with 2 mL of 20% (*w/v*) solution of the polymer. The mixture was incubated for 5 h at 95 °C, washed with Milli-Q water with subsequent centrifugation three times and resuspended in Milli-Q water for further modifications.

Proteins were conjugated with the polymer-coated nanoparticles by means of formation of covalent amide bounds using standard carbodiimide bioconjugation techniques [29]. The 600 μg of coated HNPs were incubated in 40 μL of the MES buffer (pH 5.0) for 30 min with 7 mg of EDC and 3.5 mg of sulfo-NHS. Then, the HNPs were centrifuged and washed with MES. Finally, the HNPs were washed with Milli-Q water and mixed with 40 μL of 1 mg/mL protein solution in PBS. The mixture was incubated overnight at RT. Finally, the HNPs were centrifuged and washed three times with PBS. To attach fluorescent Cy3 label to the NPs, 600 μg of protein-conjugated HNPs were dispersed in 50 μL of 0.1 % BSA solution in PBS. The solution was mixed with 17 μg of Cy3-sulfo-NHS in 50 μL PBS and was incubated for 4 h at RT.

The FIA was conducted as follows: 60 μL of 10 μg/mL protein solution in PBS was placed in a well of 96-well polystyrene plate and were incubated for 8 h at 4 °C. Then, the solution was replaced by 60 μL of 1% BSA solution in PBS (blocking buffer). After 30 min at RT, the solution was removed, and the wells were washed with 100 μL of PBS. After that, 60 μL of blocking buffer with different concentrations of the HNPs conjugated with Cy3-labeled proteins were added into the wells and were incubated for 30 min at RT. Finally, the wells were washed five times with 100 μL of PBS and Cy3-caused fluorescence was measured by ClarioStar microplate reader (BMG LABTECH, Durham, NC, USA).

Freshly harvested CHO (0.25 million), BT-474 (0.25 million) cells, and RBCs (final concentration of 0.5%) were incubated in 50 µL PBS with 3 µg of polymer-coated HNPs or 10 µg protein-conjugated polymer-coated HNPs for 10 min and washed twice with PBS. No fluorescent staining of the cells was used to minimally affect the cell–nanoparticle interactions. The RBCs were studied with an imaging flow cytometer ImageStream X Mark II (Luminex Corporation, Austin, TX, USA) using a 488-nm (50 mW) laser for the excitation of fluorescence. Herceptin was used as a negative control in the experiments with the RBC. The CHO and BT-474 cells were studied with a flow cytometer CellStream (Luminex Corporation) using 561-nm (1 mW) laser. For the ImageStream data, we used the manufacturer-recommended gating strategy [33,34,35,36,37,38]. Specifically, a histogram of the brightfield (BF) and channel gradient root mean square (RMS) values were used to discriminate between unfocused and focused images. The gradient RMS feature measures the sharpness quality of an image by detecting large changes in pixel values in the image [37]. The objects with better focus have higher gradient RMS values, thus we gated events with gradient RMS values greater than 35 during acquisition. Then, to discriminate a single cell population, the area BF feature based on the size of the object in square microns was plotted against the aspect ratio BF feature, calculated as the ratio of the object’s minor axis over its major axis [38]. Moreover, the single cells were gated so that NPs aggregates with a lower area BF value, were excluded from further analysis, as shown in Figure S8a. For the CellStream data, as the first step, we applied an area FSC vs. area SSC dot plots to identify cell population. Then, an area FSC vs. aspect ratio FSC dot plot was used to gate a single cell population, as shown in Figure S8b.

Fluorescent optical tomography was performed on a VerumSight bioimaging system (Abisense, Moscow, Russia) using a 750 ± 20 nm light source for excitation and 800 nm long-pass emission filter. 2 mg of HNPs@PAA were conjugated with total human IgG and then were labeled with 50 μg Cy7.5-sulfo-NHS dye, as described above (for Cy3). Each injected dose contained 500 μg of the NPs in 300 μL of saline solution [39]. For the experiment were used female BALB/c mice (ca. 20 g). The mice were euthanized 40 min after the injection.

Scanning electron microscope (SEM) and a transmission electron microscope (TEM) were used to acquire morphological and structural information of the nanoparticles. The SEM images were obtained with a MAIA3 microscope (Tescan, Brno, Czech Republic) at an accelerating voltage of 10 kV. The samples for the SEM imaging were diluted down to appropriate concentrations, placed onto a silicon wafer and then allowed to dry at the ambient conditions. The TEM imaging and the EDX analysis of the nanoparticles were performed on a 200 kV JEM 2100 TEM (JEOL, Tokyo, Japan). The synthesized nanoparticles were collected on the TEM copper grids with thin carbon film. The IR spectra were recorded on a FT-801 spectrometer (Simex, Novosibirsk, Russian Federation) and a iS50 FTIR spectrometer (Thermo Nicolet, Waltham, MA, USA) with attenuated total reflectance (ATR) head. The XRD patterns were collected on a SmartLab diffractometer with CuKα radiation (Rigaku, Tokyo, Japan). The DLS analysis was performed on a Photocor Complex (Photocor Ltd., Moscow, Russia) in Milli-Q water at RT. The magnetic behavior of the NPs was studied using an original MPQ technique described previously [25,40] and, briefly, based on the nonlinear magnetization of superparamagnetic nanoparticles subjected to an alternating magnetic field at two frequencies and registration of magnetic signal on their linear combination.

## 4. Conclusions

The reaction of two-line ferrihydrite with different concentrated acids was investigated. Surprisingly, the anticipated full dissolution of the hydrated oxide with the formation of the soluble salts occurred only in several cases. The reaction appears to be more complex and proceeds with only partial dissolution along with the parallel formation of hematite nanoparticles. The yield of the NPs rose with an increase of the dehydrating ability of the acid mixture. The reaction can be easily scaled up to produce considerable quantities of nanoparticles with the yield up to 10%. The resulting nanoparticles were modified with different organic polymers and used for successful in vitro cancer cell targeting after biomodification. Therefore, the reported NPs are promising material for development of functionalized nanoparticles for different biomedicine applications. Since the reaction of ferrihydrite with concentrated sulfuric acid leads to partial formation of hematite nanoparticles, the reported observation could help in the search for new pathways of mineral evolution on other planets where such interactions may occur, for example, on Mars during periods of asteroid impacts or on Venus where the sulfuric acid exists in the atmosphere. Moreover, the development of this novel procedure for the preparation of hematite NPs could lead to a discovery of new synthetic ways to other metal oxide nanoparticles.

## Supplementary Materials

The following are available online at https://www.mdpi.com/1420-3049/25/8/1984/s1, Figure S1: Experimental setup, Figure S2: TEM images of nanoparticles with different shape and morphology, Figure S3: EDX analysis of the NPs, Figure S4: EDX analysis of hematite prepared by a well-established procedure, Figure S5: TEM and SAED images of nanoparticles fabricated using different concentrated acids, Figure S6: Size distribution of the NPs synthesized using different acids, Figure S7: Hydrodynamic radii of synthesized nanoparticles according to DLS analysis, Figure S8: Gating strategy.
