# Supplementary material for: Hematite Nanoparticles from Unexpected Reaction of Ferrihydrite with Concentrated Acids for Biomedical Applications
Source: Molecules. 2020 Apr 23;25(8):1984. doi: 10.3390/molecules25081984 (PMC7221743; doi:10.3390/molecules25081984)
Supplement: Supplementary file 1 [file molecules-25-01984-s001.pdf]

molecules

Supplementary Materials

Hematite Nanoparticles from Unexpected Reaction of Ferrihydrite with Concentrated Acids for Biomedical Applications

Afanasy V. Lunin [1], Anna A. Lizunova [1], Elizaveta N. Mochalova [1,2], Maria N. Yakovtseva [1], Vladimir R. Cherkasov [1,2], Maxim P. Nikitin [1,2,3] and Eugene L. Kolychev [1,2,*]

1 Moscow Institute of Physics and Technology, 9 Institutskiy per., Dolgoprudny, Moscow Region, 141700, Russia; afanasy.lunin@phystech.edu (A.V.L.); anna.lizunova@gmail.com (A.A.L.); mochalova@phystech.edu (E.N.M.); mariantes@yandex.ru (M.N.Y.); v_r_cherkasov@list.ru (V.R.C.); max.nikitin@phystech.edu (M.P.N.)

2 Prokhorov General Physics Institute of the Russian Academy of Sciences, 38 Vavilova St., Moscow, 119991, Russia

3 Shemyakin–Ovchinnikov Institute of Bioorganic Chemistry, Russian Academy of Sciences, Miklukho-Maklaya St., 16/10, Moscow, 117997, Russia

* Correspondence: kolychev.el@mipt.ru

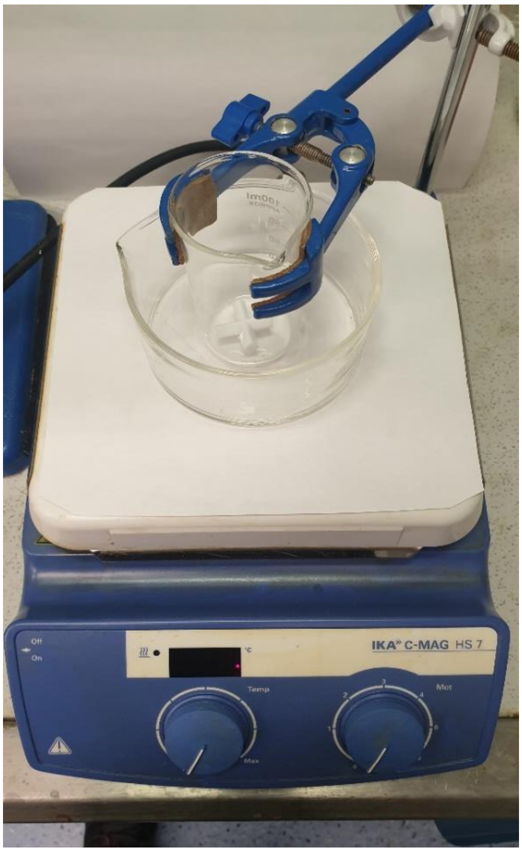

Figure S1. Experimental setup.

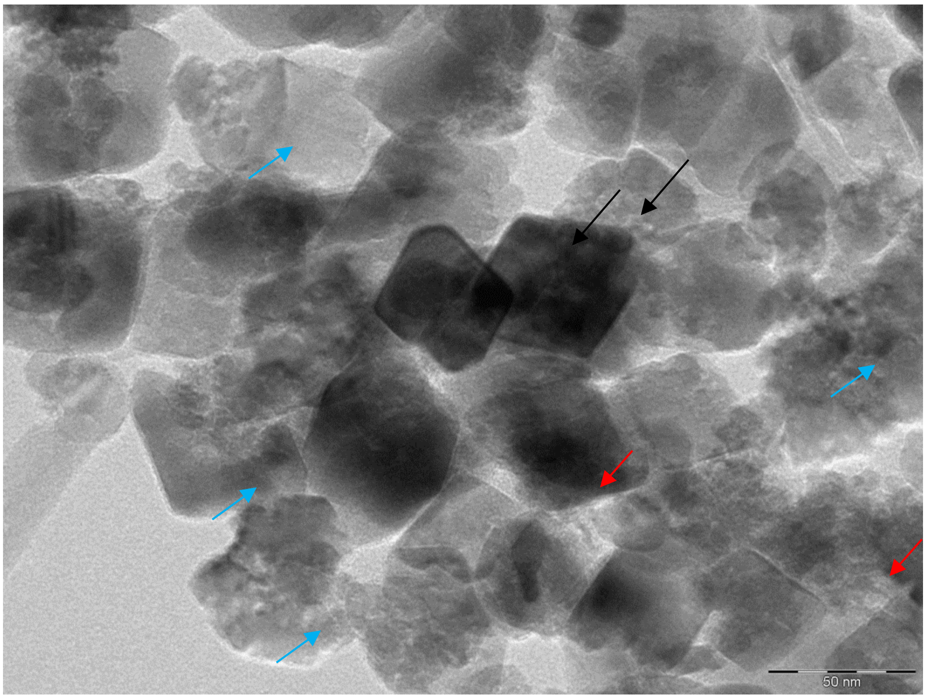

Figure S2. TEM images of nanoparticles with different shape and morphology: hexagonal bipyramid (black arrows) and rhombohedral shapes (red arrows), particles with full or partly irregular surface morphology (blue arrows).

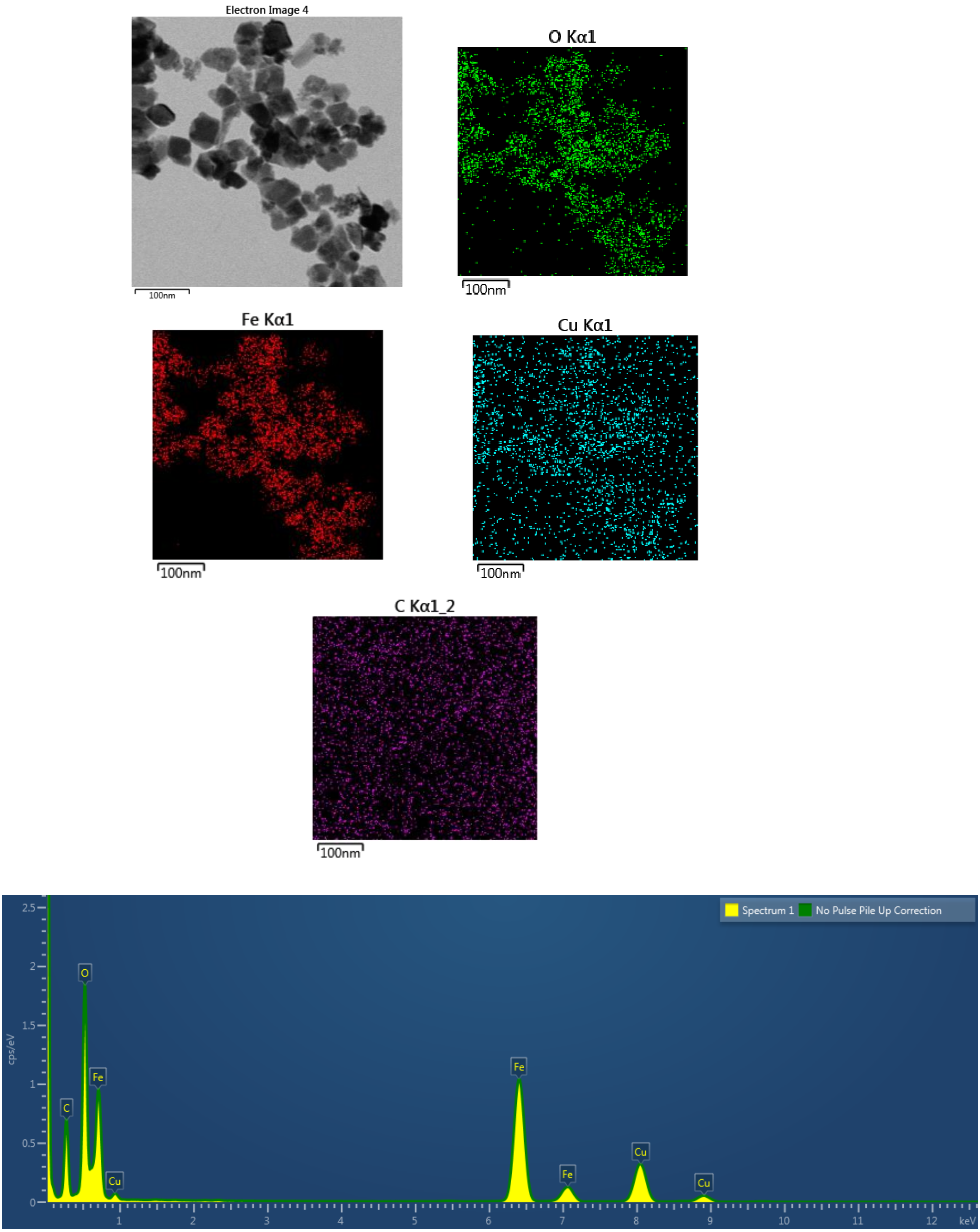

Figure S3. EDX analysis of the NPs. ~Distribution of the elements is shown by different colors: O – green, Fe – red, Cu – blue, C – purple (each insert is additionally signed on top). Peaks of carbon and copper are from the TEM grid.

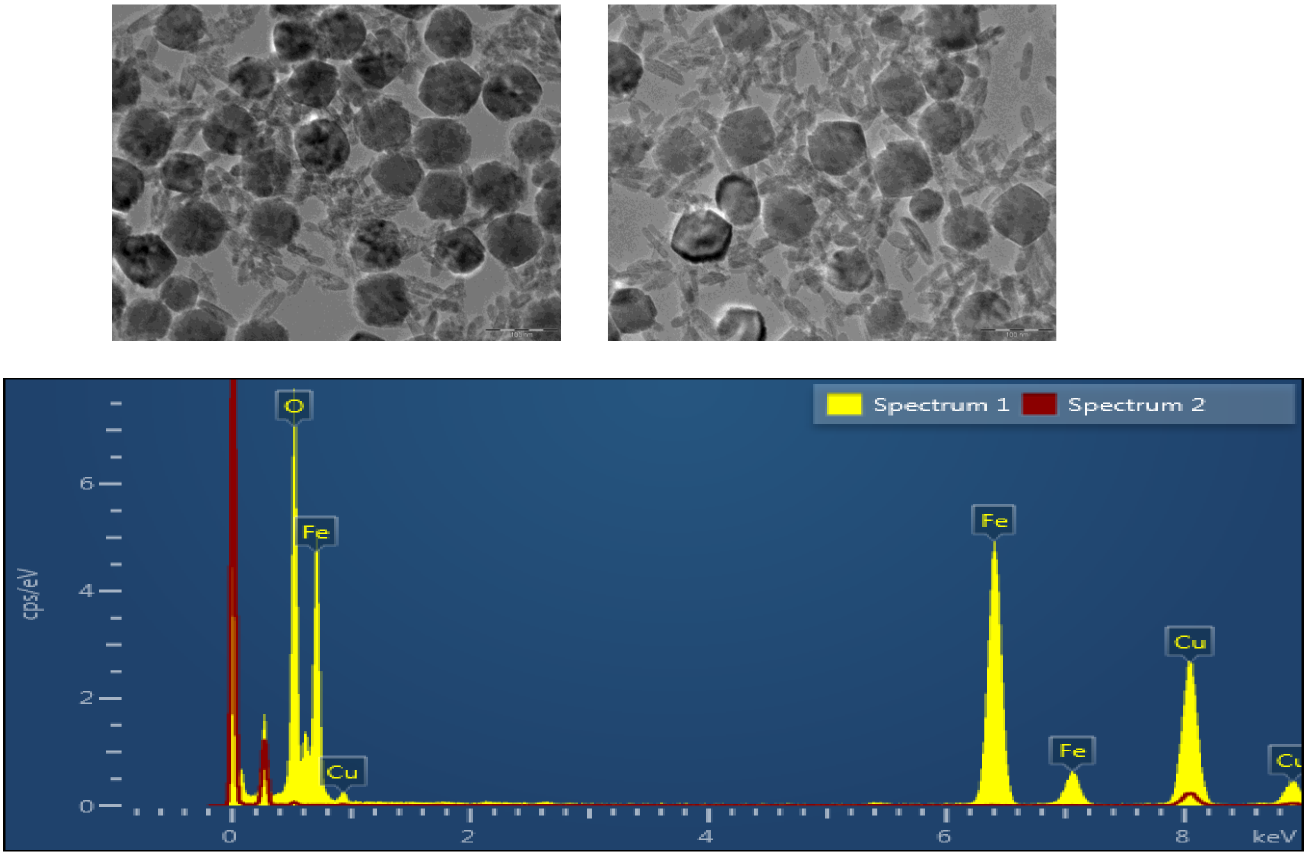

Figure S4. EDX analysis of hematite prepared by a well-established procedure.

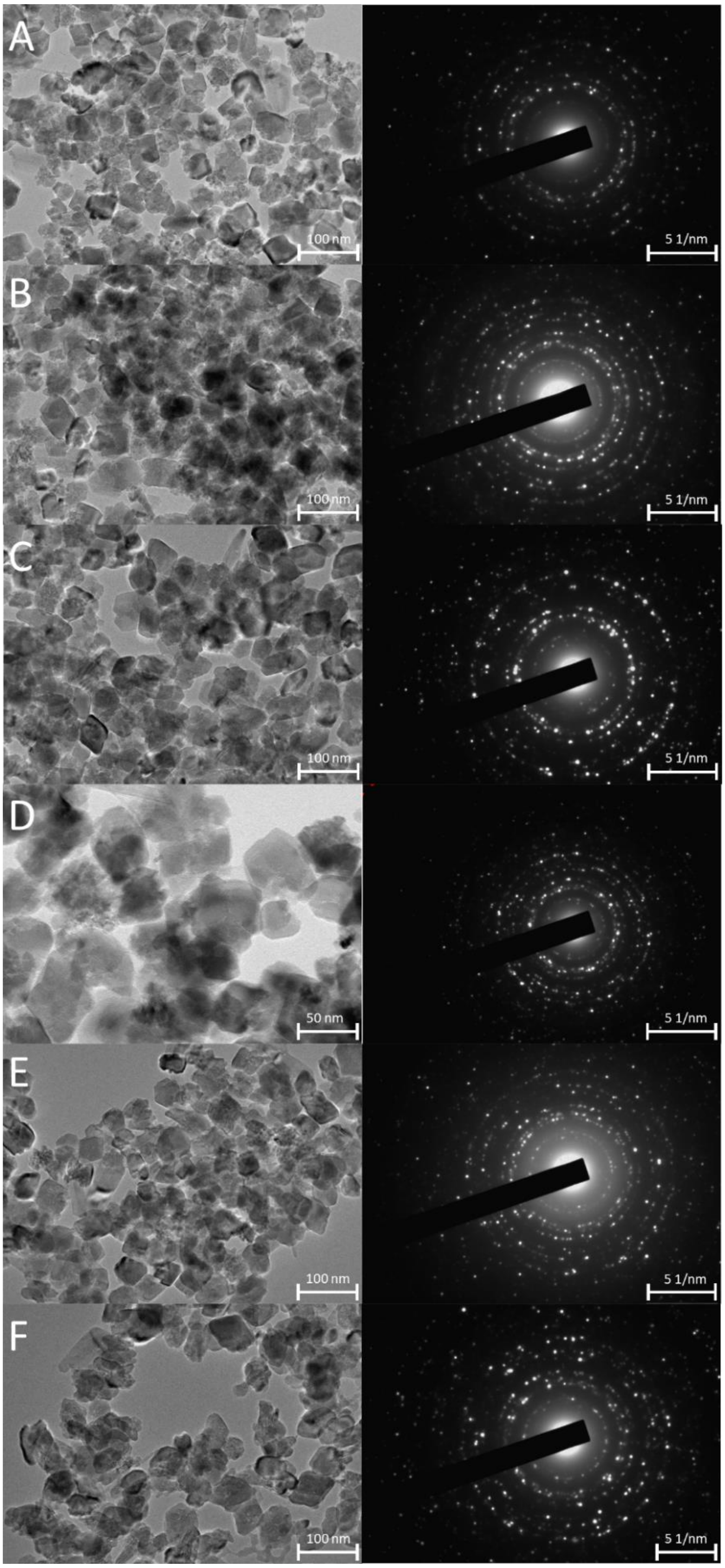

Figure S5. TEM and SAED images of nanoparticles fabricated using different concentrated acids: (**a**) Acetic acid; (**b**) Nitric acid; (**c**) Hydrochloric acid; (**d**) Ssulfuric acid; (**e**) Phosphoric acid; (**f**) Sulfuric acid and phosphorus pentoxide.

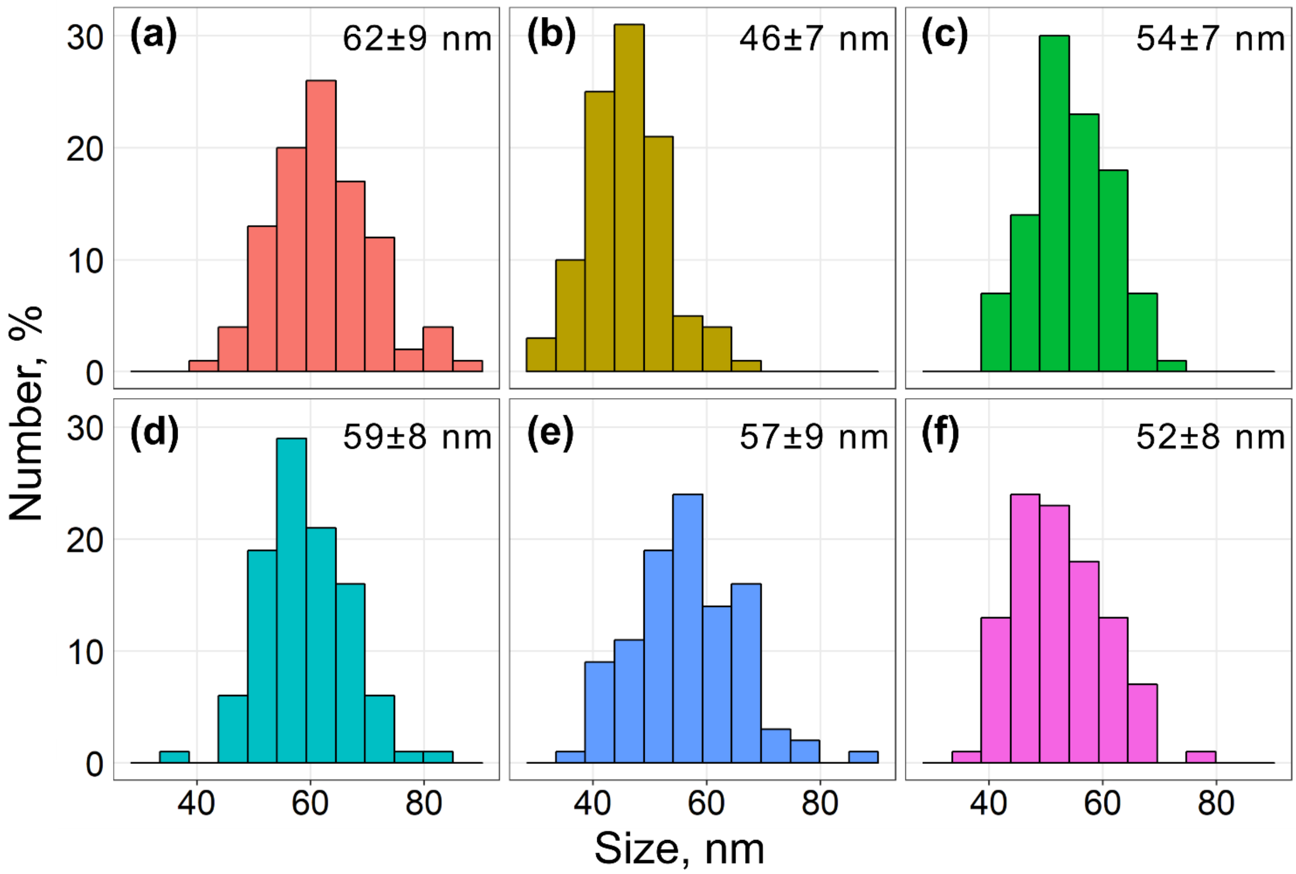

Figure S6. Size distribution of the NPs synthesized using different acids: (**a**) Sulfuric acid; (**b**) Nitric acid; (**c**) Acetic acid; (**d**) Phosphorus acid; (**e**) Hydrochloric acid; (**f**) Mixture of sulfuric acid and phosphorous pentoxide. The size was measured using SEM images; each NP's size was calculated as average of its maximum and minimum size. The values are presented as mean ± standard deviation (n=100). The images were processed using the Fiji/ImageJ software.

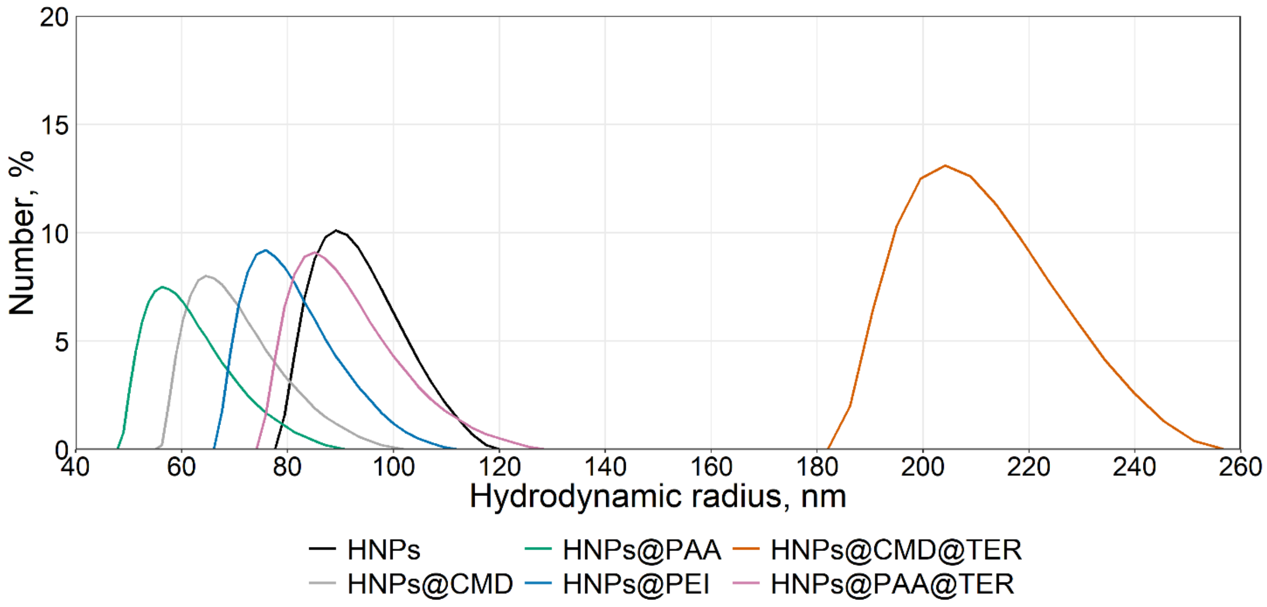

Figure S7. Hydrodynamic radii of synthesized nanoparticles according to DLS analysis.

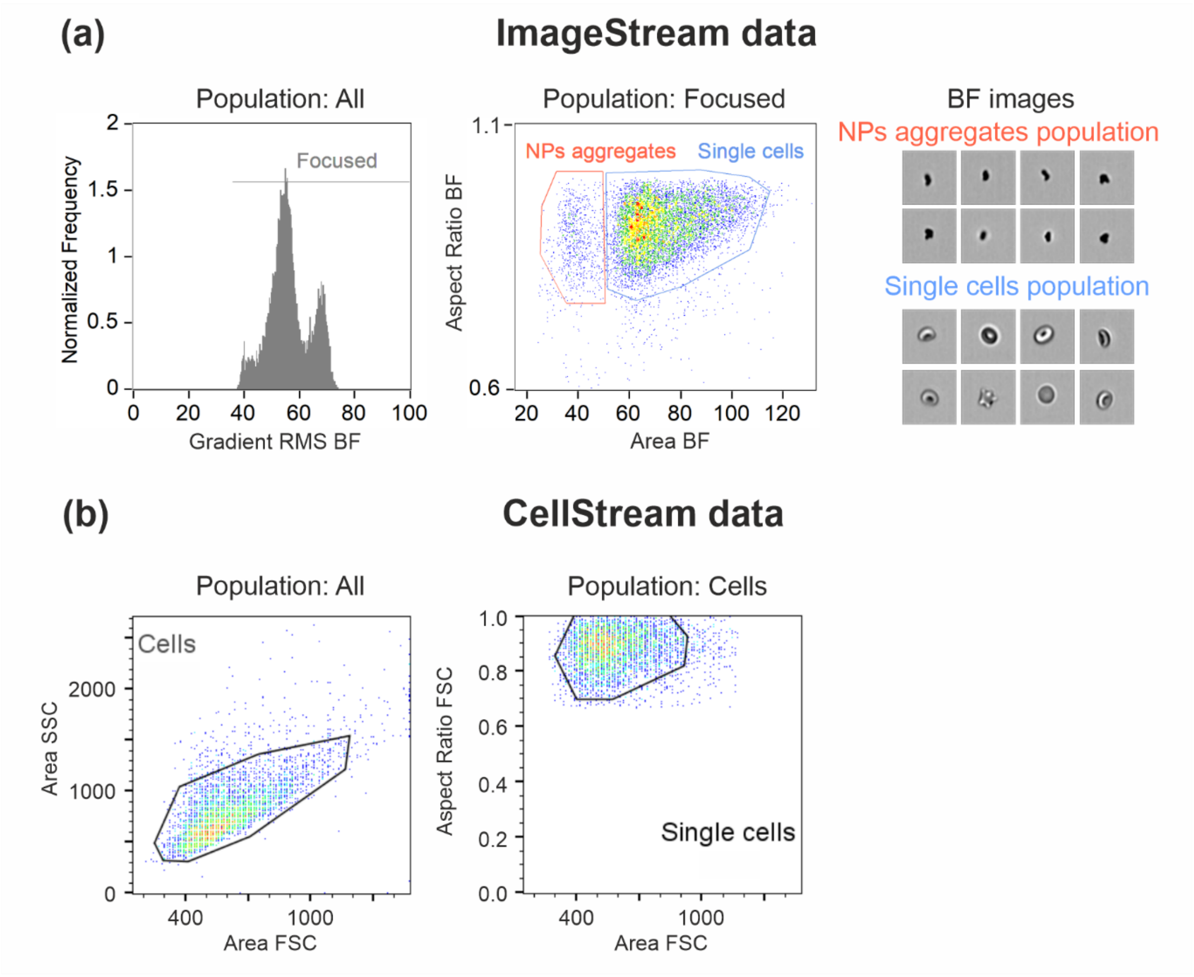

Figure S8. Gating strategy. (**a**) For the ImageStream flow cytometer experiments with erythrocyte targeting (an example is shown for the RBCs with HNPs@CMD@Control Ab); (**b**) For the CellStream flow cytometer experiments with BT-474 and CHO cell targeting (an example is shown for the BT-474 cells with HNPs@PAA@Trastuzumab-Cy3).
